# Protocol for a Delphi consensus study to identify priority characteristics of integrated care for individuals with severe mental illness and comorbid physical disorders in Europe

**DOI:** 10.1371/journal.pone.0352089

**Published:** 2026-07-09

**Authors:** Esther Touitou-Burckard, Tomasz Gondek, Ulker Isayeva, René Ernst Nielsen, Heidi Taipale, Jari Tiihonen, Laurent Boyer, Coralie Gandré, EU-MIND Consortium Collaborators

**Affiliations:** 1 Assistance Publique – Hôpitaux de Marseille, Research Centre on Health Services and Quality of Life (CEReSS), Aix Marseille University, Marseille, France; 2 Institute for Research and Information in Health Economics (Irdes), Paris, France; 3 Institute of Social Studies, University of Lower Silesia, Wroclaw, Poland; 4 Section of Psychiatry, Department of Medical Science and Public Health, University of Cagliari, Cagliari, Italy; 5 Aalborg University Hospital, Department of Clinical Medicine, Aalborg, Denmark; 6 Aalborg University, Faculty of Medicine, Psychiatry, Aalborg, Denmark; 7 Department of Forensic Psychiatry, University of Eastern Finland, Niuvanniemi Hospital, Kuopio, Finland; 8 Department of Clinical Neuroscience, Karolinska Institutet, Stockholm, Sweden; 9 Centre for Psychiatry Research, Stockholm Health Care Services, Region Stockholm, Stockholm, Sweden; 10 Fondation FondaMental, Créteil, France; University of Malaga: Universidad de Malaga, SPAIN

## Abstract

**Introduction:**

Individuals with severe mental illness (SMI) experience persistent and complex physical health needs that remain insufficiently addressed. While integrated care represents a promising solution, there is no consensus among stakeholders regarding what constitutes best-practice organizational models for this population. As part of the European Mental and Physical Health Initiative for People with Severe Mental Disorders (EU-MIND), this study aims to identify expert consensus on the key characteristics of integrated care models for individuals with SMI to support their sustainable implementation across Europe.

**Methods:**

This study will use an online Delphi process, with up to three rounds, to engage stakeholders from six European countries (Denmark, Finland, France, Italy, Poland, and Sweden). Participants will include people living with SMI, their relatives, health and care professionals, public decision-makers and institutional actors with relevant experience related to the research topic. A minimum sample size of 33 participants per country will be targeted, with the aim of ensuring balanced representation across the different categories of participants. They will be asked to rate the importance of potential key characteristics of integrated care models using Likert scales. A characteristic will be considered to have reached consensus if more than 70% of the respondents agree on its degree of importance. This study complies with the Delphistar reporting guidelines for Delphi studies and has received ethical approval from the Aix-Marseille University Ethics Committee and the Swedish Ethical Review Authority.

**Discussion:**

This study will provide expert-based guidance on the core characteristics of integrated care for individuals living with SMI. By capturing diverse stakeholder perspectives across countries and healthcare systems, it will help define shared priorities and inform future service design, implementation and policy, supporting sustainable and context-sensitive care development in Europe.

## Introduction

Severe mental illnesses (SMIs) are chronic conditions that include psychotic, bipolar and major depressive disorders. Such illnesses significantly impair daily functioning, particularly professional and social activities [1,2]. From a health system perspective, individuals with SMI constitute a high-need, high-cost group [3,4], not solely because of the mental healthcare they require [5]. Their physical health needs are often complex, with disproportionately high rates of comorbidities, such as cardiovascular and respiratory disorders [6–9]. People with SMI have a life expectancy 15–20 years shorter than that of the general population, mainly attributable to preventable somatic causes [10]. This suggests a systemic failure of health systems to address these multiple comorbidities effectively, notwithstanding international conventions supporting the right of all individuals to enjoy the highest attainable standard of physical and mental health [11].

Individuals with SMI experience poorer physical health outcomes due to a complex interplay of factors. These include unhealthy lifestyle behaviors (e.g., physical inactivity, poor diet, and tobacco use), adverse effects of psychiatric medications on physical health (e.g., weight gain, insulin resistance or dyslipidaemia), social isolation, unstable housing conditions, and inadequate healthcare access and delivery [12,13]. Stigma and diagnostic overshadowing, which is the tendency to attribute all somatic complaints to manifestations of the SMI, are hypothesized to contribute to delays in the diagnosis of physical health conditions [14]. Another key contributor is the lack of integration across care settings. Evidence shows major fragmentation in coordination between primary and secondary care, between physical and mental health services, and across health and social care systems [15–17]. Preventive services – such as cancer screening and cardiovascular risk management – are particularly difficult for individuals with SMI to access [6,18,19]. Furthermore, guideline-concordant care is often underused in this population across many countries [6,7,20–23].

The World Health Organization (WHO) has recently emphasized the importance of holistic care in its mental health system guidelines, highlighting physical health as a key priority for individuals with SMI [24]. A promising strategy to address the persistent health inequalities in this population is the implementation of integrated care programs. These programs involve coordination across levels of care, shared planning, and interprofessional collaboration to improve both health outcomes and patient experience while reducing costs [25,26]. Such approaches are particularly beneficial for individuals with comorbid chronic conditions [27], as multimorbidity challenges traditional siloed, disease-specific care frameworks. Several integrated care models have been developed for individuals with SMI that address mental health, physical conditions and, in some cases, the broader social needs associated with complex life trajectories [28].

However, no country has succeeded in sustainably delivering global integrated care for this specific population. Existing programs often fail to scale beyond the pilot stage and to achieve system-wide adoption. Evidence regarding their effectiveness remains mixed, and there is substantial variability in their characteristics – particularly with respect to their organizational components [28]. Moreover, the involvement of individuals with SMI in the design of integrated care models is rare, despite being critical to ensuring the relevance of such models and their responsiveness to actual needs [17,24,29,30]. To date, there is no global consensus among key stakeholders regarding what constitutes best-practice integrated care for this population. This lack of agreement hinders collective efforts to scale up successful initiatives beyond pilot projects and limits the transferability of models across countries with different health systems and policies.

In this context, this study aims to identify expert consensus on the key characteristics of integrated care models for people living with SMI. The study will draw on the perspectives of diverse stakeholder groups across Europe. It is conducted as part of the European Mental and Physical Health Initiative for People with Severe Mental Disorders (EU-MIND, https://eumind.eu/), funded by the 2024 European Partnership on Transforming Health and Care Systems. EU‑MIND is a three‑year interdisciplinary consortium involving Denmark, Finland, France, Italy, Poland, and Sweden. Its aim is to develop a European solution to improve the organization of care for people with SMI and co‑occurring physical disorders. Unlike initiatives targeting specific comorbidities, EU‑MIND adopts a comprehensive approach to health encompassing all somatic conditions relevant to individuals with SMI. The project is structured into several work packages covering: (1) analysis of nationwide health claims databases to identify gaps in care pathways and mortality outcomes; (2) an umbrella review of existing integrated care programs for people with SMI; (3) the Delphi consensus process reported in this article, focused on identifying priority characteristics of integrated care to support; (4) a health‑economic evaluation of societal attitudes toward implementing these priority features and its financial impact; and (5) a feasibility pilot of such implementation in real‑world settings. A dedicated dissemination work package also supports knowledge translation across Europe.

## Materials and methods

### Setting

The study will be conducted in the six European countries participating in the EU-MIND initiative: Denmark, Finland, France, Italy, Poland, and Sweden. These countries were selected to reflect a diversity of geographical regions – Northern, Southern, Western, and Eastern Europe – as well as a range of health system models. These models include choice- and supply-oriented systems based on social insurance (France); performance- and primary-care-oriented public systems (Finland and Sweden); regulation-oriented public systems, particularly with respect to healthcare access (Denmark and Italy); and low-supply, low-performance mixed systems (Poland) [31].

The study will be conducted by an interdisciplinary research team from these six countries, bringing together health services researchers, epidemiologists, clinicians, and neuroscientists who have been working extensively on physical health issues for people with SMI (e.g., [6,10,23,32–37]).

### Study design

To identify, refine and establish a consensus among a panel of experts, we will conduct a Delphi study. The Delphi method has been increasingly and widely employed in recent health sciences research as a systematic approach to gathering expert judgement and discerning areas of agreement and disagreement where high-quality evidence is limited [38]. This method is grounded in principles designed to reduce biases commonly encountered in group discussion, particularly those arising from conformity and authority, while facilitating the reassessment and refinement of judgements [39]. Key methodological features include the anonymous participation of individuals with specialized knowledge on the topic of interest, a structured communication process based on standardized iterative questionnaires, the provision of controlled feedback on the panel’s responses between each iteration with the presentation of interim results, and the statistical analysis of the responses obtained [40,41].

Our study will use an online Delphi process (eDelphi), with up to three rounds, to engage experts from various European countries, thus overcoming geographical barriers, ensuring anonymity, and enabling timely data collection [42].

### Sampling and participants

This study will involve a diverse group of participants, who represent the main stakeholders affected by the development of integrated care for SMI. These stakeholders will include people living with SMI, their relatives, health and care professionals, public decision-makers and institutional actors. Participants may belong to more than one of these categories. To ensure the heterogeneity of the expert panel, which is essential to the quality and relevance of the consensus reached [43], several subcategories within each stakeholder group will be considered (Table 1).

**Table 1:** Number of targeted experts by category and country.

Category*	Subcategory	N
**People with lived experience of SMI**	People living with SMI (e.g., psychotic, bipolar or major depressive disorders)	3
	Relatives of people living with SMI	3
	Peer supporters	3
**Health and care professionals**	Psychiatrists (e.g., working in a hospital or private practice)	3
	Psychologists (e.g., working in a hospital or private practice)	3
	Somatic physicians (e.g., general practitioner, emergency physician, other medical specialist)	3
	Nurses (e.g., working in a hospital or private practice)	3
	Social workers	3
**Public decision-makers and** **institutional actors**	At the local level (e.g., hospital management or municipal health agency)	3
	At the regional level (e.g., regional health agency)	3
	National administration (e.g., Ministry of Health, Directorate of Mental Health Policy)	3

* Categories are not mutually exclusive (e.g., a peer supporter with lived experience of SMI can also be a care professional in some countries; health professionals or public decision-makers may themselves have lived experience of SMI…). Participants will be free to self-identify as they choose when completing the questionnaire.

The Delphi method uses nonprobability sampling to recruit participants. Experts will be identified by national research team members, drawing on their well-established networks developed through prior work in this field. Eligible participants must have relevant expertise, either through lived experience of SMI – including personal use of mental health services, engagement on behalf of a relative, or drawing on their experience to support others with similar issues – or through professional experience working in services, organizations, or systems supporting individuals with SMI. In addition, eligible participants must show willingness and ability to contribute meaningfully, have the capacity to offer their consent to participate, be available throughout the study, and have adequate communication skills. Subcategories may be further refined by national teams to account for country-specific contexts. For example, in countries where municipalities are responsible for healthcare provision, it will be important to include decision-makers at this level, an adaptation that may be less relevant elsewhere. Only individuals aged 18 or over will be invited to participate.

To increase the representativeness of the expert panel, efforts will be made to achieve balance in terms of gender, stakeholder subcategories, and country of residence within the limits imposed by the availability and willingness of potential participants. In the context of heterogeneous samples, there is no clear consensus on the optimal size of a Delphi panel, although a minimum of 5–10 experts per type of expertise across the entire sample is generally recommended [44]. The expected sample size per country was therefore determined collaboratively by the research team on the basis of a balance between the need for diverse perspectives and practical constraints. The targeted number of experts per category and country is detailed in Table 1. We aim to recruit a minimum of 33 participants per country, for a total minimum sample of 198 participants across all six countries. To mitigate the risk of nonresponse, additional individuals per stakeholder category will be contacted. Attrition may disproportionately affect certain stakeholder groups, particularly individuals living with SMI. Specific retention strategies will therefore be implemented. They include a detailed explanation of the iterative nature of the Delphi process prior to the first round, personalized email communications at all stages of the study, concise questionnaires designed to minimize participant burden, systematic reminders procedures, and flexible survey completion options. For participants with SMI, this flexibility includes the possibility of completing questionnaires with the assistance of a trusted relative or caregiver. In addition, adjustments to the study timeline will be proactively communicated to participants to support continued engagement across rounds. Any difficulties encountered during the course of the study will be addressed and resolved collaboratively, and will be transparently documented in the final study, in line with recent guidelines [40].

### Survey questionnaire content

The Delphi survey will consist of up to three rounds of online questionnaires. The first questionnaire will collect nonidentifying demographic information regarding the participants, including age group, gender, main country of residence and category(ies) of expertise. For health and care professionals, additional data will be collected regarding their type of profession, while for public decision-makers and institutional actors, information regarding their level of action (local, regional or national) will be gathered. Participants will then be presented with a series of potential characteristics of integrated care models that could be prioritized to improve overall care for individuals with SMI. They will be asked to rank the importance of each characteristic using a numeric Likert scale ranging from 1 (not important) to 9 (very important), a format commonly used to measure continuous constructs [45]. Despite the absence of an explicit ‘do not know’ or ‘prefer not to answer’ response option, participants will be instructed to use the midpoint value of the scale (score = 5) to indicate uncertainty or insufficient knowledge regarding a given proposal. The closed-ended questions will be organized into thematic sections corresponding to six key dimensions of integrated care for multimorbidity, as defined by previous conceptual research. These dimensions include governance, workforce, service delivery, funding, information and technology, and research and evaluation [27]. Each question will focus on a specific area of action that could be incorporated into integrated care models for SMI. Several proposals describing care components or actionable measures aimed at supporting integrated care for people with SMI will be presented (Table 2). These proposals were empirically derived from an umbrella review of the literature on existing integrated care programs addressing SMI and multimorbidity [28]. Their applicability across diverse national contexts was further validated through internal consultations with members of the EU-MIND consortium. Finally, the questionnaire will include an open-ended question inviting participants to share one essential change they would make to the organization of care for people with SMI to identify an ultimate priority among those presented. The answer to this question may also be used by participants to propose additional topics for discussion or to provide commentary.

**Table 2:** Overall content of the questionnaire.

Dimensions	Questions	Propositions
**Governance**	**Emergence of the model**	Bottom-up (developed by health or care professionals) Top-down (developed by decision-makers or institutional actors)
		Implication of service users and relatives in model design
		Local to national
		National to local
	**Scope of the model**	Specifically dedicated to health and care support for people with SMI
		Dedicated to health and care support for all chronic diseases
	**Global governance of the model**	Shared vision and commitment about coordination and integration of services
		Commitment of the administrative and executive personnel to the integration of care
		Clear guidelines for the roles, responsibilities and collaboration of all involved professionals
		Care in line with national and international best practice recommendations
**Service delivery**	**Care setting**	Primary care
		Outpatient mental healthcare
		Inpatient mental healthcare
		Social care
		Dedicated facilities
	**Degree of integration**	Collaboration at distance
		Collaboration on site
		Full collaboration in a colocated setting
	**Person-centred approach**	Care provision considers socioeconomic vulnerabilities
		Care provision is culturally sensitive
		Self-management training for service users and relatives
		Shared case reviews and decision-making, including service users
		Coordination tailored to complexity
	**Continuity of care**	Regular follow-ups and screenings
		Focus on medication side effects, interaction, and adherence
		Practical assistance to facilitate access to care
		Home visits
		Access to care at out-of-office hours
**Workforce**	**Team-based care**	Multiple medical specialties
		Multiple types of health professionals
		Involvement of social workers
	**New professional roles**	Case/care manager
		Advance nurse practitioner
		Peer-worker
		Medical assistant
	**Professional training**	Team-based care and coordination training
		Physical health training for mental health professionals
		Training in mental health and on specific needs of people with SMI for physical health professionals
		Common training between mental and physical health professionals
		Training on social representations of mental illness, including destigmatization initiatives
**Funding**	**Global funding model**	Free access to integrated care for service users
		Payment equity and attention towards fair payment models
		Stimulating investments in innovative care
		Specific budget dedicated to care coordination and integration within care organizations
	**Financial incentives for coordination**	Pay for coordination
		Bundled payments
		Pay for performance
		Population-based payments
	**Financial incentives for service users**	No out-of-pocket payment
		Direct financial incentives
		Free training and workshops
**Information and technology**	**Information system**	Shared data systems among professionals
		Interoperable data systems and exchange of information between professionals
		Patients’ portal: users can access their own data
		Data ownership and protection policies: users may object to the sharing of certain information
		Specific financial support for numeric innovation
	**Use of new technologies**	Tele-medicine
		E-health tools for service users
		E-health tools for professionals
		Assistive technologies
		Remote monitoring
	**Collection of data**	Data on service users and their relatives’ experience and satisfaction
		Longitudinal individual health data to monitor evolution and adapt care
		Development of algorithms to predict individual health risks
		Registries to support a population health approach
**Research and evaluation**	**Overall model evaluation**	Impact on service users’ health, experience and quality of life
		Impact on health and care professionals’ practices
		Evaluation of model implementation
		Participatory evaluation, including service users and their relatives
	**Dimensions of model effectiveness for service users**	Increased access to physical healthcare
		Improved physical health outcomes
		Improved mental health outcomes
		Reduced social health-related needs
		Service users and their relatives’ satisfaction and quality of life

The Delphi questionnaire will be identical for all participants, regardless of their category (or categories) of expertise. To ensure inclusivity and accessibility, it will be formulated using language that is easily understood by the general public, with explanations and definitions provided where necessary to clarify technical terms or complex concepts. Researchers from the EU-MIND consortium will review and pilot test the questionnaire prior to the first round. The questionnaire will also be pilot tested by individuals with lived experience of SMI, lay members of the general population with no prior involvement in the project, and informed experts external to the research team. Pilot testing will assess completion time, the overall clarity of the study aims, and the wording and internal coherence of questionnaire proposals, with particular attention to accessibility of language. Feedback from this process will be used to revise the questionnaire prior to its implementation in the Delphi rounds. Each questionnaire will be translated into the six native languages of the participants using a forward-backward machine translation process combined with a human quality check [46]. Machine translation will rely on standard translation services such as DeepL, in combination with ChatGPT OpenAI’s advanced language model. A human quality check will be conducted by national team members who are bilingual in both English and the target language, ensuring the cross-cultural validation of the questionnaire content. The estimated time to complete the full initial questionnaire will be around 20 minutes.

Within the questionnaire, propositions that will have reached consensus in terms of their importance or their lack of importance (see Analysis section) will be excluded from subsequent rounds of the Delphi survey. Propositions for which consensus is not reached, or that are rated as of uncertain importance, will be reassessed in the following round to specifically explore contentious areas and existing disagreements related to the topic under study. The second round may also incorporate new propositions suggested by participants through the open-ended question of the first round. Additional propositions will be included if they meet the following criteria: their topic is relevant to integrated care for individuals with SMI and comorbid physical conditions; they are sufficiently clear and actionable to be operationalized as Delphi items; they are not redundant with propositions already included in the questionnaire. To support accurate interpretation and translation of propositions submitted in different languages, country representatives will be consulted as needed. However, final decisions regarding inclusion will remain the responsibility of the French team, in charge of the Delphi coordination, to ensure methodological consistency across countries.

### Study process

The recruitment process in each country will begin with an initial email sent by national team members. This email will briefly introduce the Delphi survey and provide a unique link to an electronic survey platform where the email recipient will be able to review a participant information statement and access an online consent form. The participant information statement will explain the purpose of the study, the criteria for participant selection, and the participants’ role in the research. It will also describe the survey methodology, including the number of questionnaires to be completed and the estimated duration of participation. Additionally, the statement will outline how the collected information may be used and provide details regarding participants’ rights, as well as any potential risks, constraints, or benefits associated with participation. It will also provide contact information for further inquiries, including for the national team members, the principal investigators, and the data protection officer. The consent form will record participants’ agreement to take part in the study and to be contacted by the research team via email, in accordance with European data protection regulations.

A few weeks after providing consent, participants enrolled in the study will receive an email containing a link to the first questionnaire. They will have a two-week period to complete and submit their responses. Participants will have the option to save their answers, enabling them to complete the questionnaire over multiple sessions if needed.

**Fig 1:**
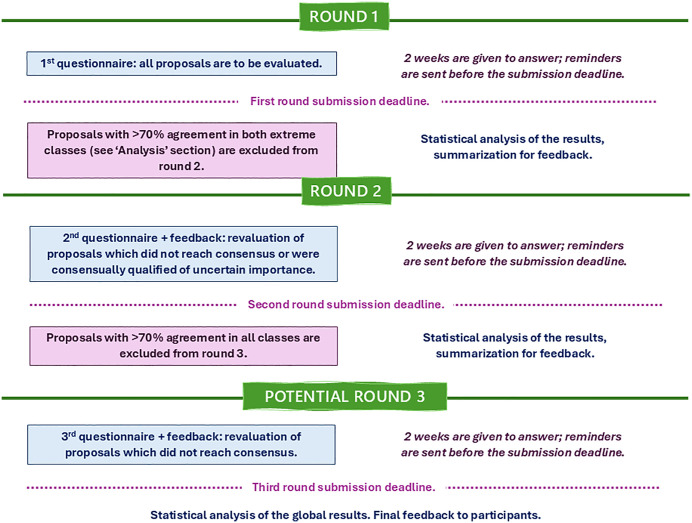
Summary of the study process.

The second questionnaire will be distributed to all the experts who will have completed the first one in the weeks following its closing date (Fig 1). The same timeline will be followed for the distribution of the third questionnaire. Participants will again have a two-week period to complete the questionnaire and will receive reminder emails if no response is received. They will also receive controlled feedback summarizing the results of the preceding round with the second and third questionnaire. This feedback will include an aggregated and anonymized summary of the responses from the entire panel, and the list of proposals regarding which consensus has been reached and which will therefore be excluded from further evaluation. This structured feedback process is designed to support informed reflection and facilitate convergence of opinion across rounds.

A third Delphi round will be conducted only if attrition remains below 40%, rating stability analyses indicate residual instability at the panel, individual or subgroup level (i.e., by country or stakeholder category) [47], and at least 25% of proposals have not reached consensus after the second round. The decision to proceed will therefore be based on the a priori assessment that an additional round is likely to generate robust and meaningful additional data to inform the final interpretation of the consensus.

The electronic questionnaires will be administered using LimeSurvey™ software (LimeSurvey GmbH, Hamburg, Germany). This platform ensures the pseudonymization of participants and their responses by storing contact email addresses separately from questionnaire data. LimeSurvey is fully compliant with the European General Data Protection Regulation (GDPR).

Overall, the entire Delphi process is expected to last approximately three months (Fig 1). Recruitment of participants started on the 20/11/2025 and will close the day before the launch of the first round, which is scheduled for the 04/03/2026. The results from the three rounds are expected to be collated in May 2026.

### Analysis

Aggregated responses will be analyzed to evaluate overall response rates, levels of agreement, mean and median values, standard deviations and interquartile ranges, percentage frequency distributions, and rankings by country and expert category. Participants will be included in all categories to which they report belonging, and analyses by expert category will therefore be conducted using overlapping groups. The number of participants contributing to each category will be reported accordingly. We will use descriptive statistics to characterize the composition of the panel of participants at each round and the percentage of dropouts between rounds. In accordance with the methodology used in prior research [45], consensus on the significant importance of a proposal will be defined as at least 70% of participants rating the proposal between 7 and 9 on the Likert scale. Conversely, consensus on the lack of importance will be defined as at least 70% of participants rating the proposal between 1 and 3. Ratings outside both extreme classes, i.e., between 4 and 6, will be interpreted as reflecting an uncertain level of importance. Even if a consensus is reached within this score range, these proposals will be retained and included in the second round of the survey for further consideration. As the third round is the final round, only proposals on which the participants have not reached a 70% consensus of any type (lack of importance/uncertain importance/significant importance) will be reassessed. Proposals that received a majority of ‘uncertain’ ratings in the first two rounds will therefore be excluded from the third round. The percentage of experts who changed their opinion, per proposal and per round, will also be calculated. With respect to the open-ended question, we will measure the percentage of experts providing additional qualitative information, and their responses will be analysed using recent methods for free-text responses in health sciences Delphi studies [48].

### Data management plan

The data collected at the end of the survey, excluding any identifying information or sensitive data, will be extracted from the LimeSurvey™ software, encrypted, password-protected, and then transmitted to the Secure Data Access Center (CASD, https://www.casd.eu), where they will be stored and processed. Access to the data will be restricted to the French research team of the EU-MIND project, which will be responsible for the Delphi data analysis in discussion with all team members involved in the survey. The extraction of individual-level data from the CASD secure environment is strictly prohibited. This system complies with the GDPR by ensuring the confidentiality and integrity of the data and enabling full traceability of access.

### Ethical considerations

This study is conducted in accordance with the ethical principles of the Declaration of Helsinki and of the Belmont Report [49,50]. Its methodology complies with the DelphiStar reporting guidelines for Delphi studies in the health and social sciences [40], and quality indicators for Delphi studies are incorporated into the design of the survey [44]. This research study has received ethical approval from the Ethics Committee of Aix-Marseille University (Approval Notice No. 2025-06-26-05 of the 26th of June 2025) and the Swedish Ethics Review Authority (Final Approval Notice 2025-08756-02 of the 19^th^ of January 2026). At any time, participants will have the right to request access to, rectification of, or restriction of the processing of their data collected in the context of the research and to withdraw their consent to its collection without having to provide justification and without consequence.

## Discussion

This study protocol describes the design and features of an online Delphi process aimed at establishing expert consensus on the key characteristics of integrated care models for individuals with SMI, thereby supporting the sustainable implementation of such models across Europe. The findings will help fill gaps in the research literature regarding best-practice organizational models for this population by engaging a broad range of stakeholders, including individuals living with SMI, their relatives, health and care professionals, public decision-makers and institutional actors from six European countries. By capturing diverse stakeholder perspectives across countries and healthcare systems, this study will identify shared priorities to guide the design, implementation, and policy development of integrated care for individuals with SMI in Europe. The proposals reaching consensus will be mapped onto key dimensions of integrated care for multimorbidity (e.g., governance, workforce, service delivery…) and translated into actionable design elements. Together, these elements will constitute the formal specification of the integrated care model which will serve as the basis for the subsequent phases of the EU‑MIND project. These phases will include a health economic evaluation, assessing societal knowledge, attitudes, practices, willingness to pay, and the financial implications of implementing this consensus‑based integrated care model for people with SMI, as well as a feasibility pilot study examining the model’s practical applicability and sustainability.

The Delphi technique is particularly suited for achieving consensus among a diverse group of experts because of its ability to ensure participant anonymity and reduce potential bias [38]. In designing this protocol, we followed recent consolidated standards for the development and reporting of Delphi studies [40,44] to ensure methodological rigor. Furthermore, the questionnaire content was informed by prior conceptual research on the core characteristics of integrated care models for multimorbidity [27], an umbrella review of the literature [28], input from members of the EU-MIND consortium who are recognized international experts in the field, and pilot testing. Nevertheless, the results of this study will have to be interpreted in light of the inherent limitations of the Delphi method [51]. Selection bias may arise from reliance on voluntary participation and from the composition of the expert panel, as participation is influenced by availability, motivation, and prior interest in the topic, potentially leading to the over‑representation of certain viewpoints. In addition, the framing and wording of questionnaire proposals may shape participants’ interpretations and responses, despite iterative refinement and pilot testing. Although anonymity and controlled feedback are intended to reduce domination effects, social desirability bias and normative pressures may still operate, particularly across rounds, as participants are exposed to aggregated group responses. Such dynamics may contribute to convergence that reflects accommodation or compliance rather than genuine changes in individual opinion. Attrition across rounds may further accentuate these effects if remaining participants hold relatively homogeneous views. While several design features of our Delphi survey were implemented to mitigate these risks, including stakeholder diversity, anonymized rating procedures, and pre‑specified stopping criteria, these biases cannot be fully eliminated and will have to be considered when interpreting the findings. Despite these limitations, by fostering consensus among diverse stakeholders, applying a rigorous and transparent methodology, and addressing a critical gap in the care of individuals with SMI, this study has the potential to significantly advance integrated care for this population across Europe, ultimately contributing to more equitable, effective, and person-centred health systems.

## Supporting information

S1 Appendix Inclusivity-in-global-research-questionnaire. (DOCX)
