# Supplementary material for: Protocol for a Delphi consensus study to identify priority characteristics of integrated care for individuals with severe mental illness and comorbid physical disorders in Europe
Source: PLoS One. 2026 Jul 9;21(7):e0352089. doi: 10.1371/journal.pone.0352089 (PMC13392973; doi:10.1371/journal.pone.0352089)
Supplement: Attachment — Submitted filename: Review manuscript PONE-D-26-08246_Comments to Authors.pdf [file pone.0352089.s002.pdf]

Dear Authors,

Thank you for the opportunity to review this manuscript.

This manuscript presents a well-designed protocol for a multinational Delphi study aimed at achieving consensus on the key characteristics of integrated care models for individuals living with severe mental illness (SMI) and comorbid physical disorders. The study is conducted under the scope of the European Mental and Physical Health Initiative for People with Severe Mental Disorders (EU-MIND) and involves stakeholders from six European countries: Denmark, Finland, France, Italy, Poland, and Sweden.

Through up to three iterative rounds, participants (including individuals living with SMI, their relatives, health and care professionals, and decision-makers) will evaluate and rate the importance of proposed key characteristics of integrated care models for this population. The questionnaire is structured around six dimensions derived from the SELFIE framework (Leijten et al., 2018). Results are expected to inform future design and implementation of integrated care models for people living with SMI and comorbid physical conditions across Europe.

The study addresses a timely and highly relevant research question. The continued gap between mental and physical healthcare for individuals with SMI is indeed a well-documented systemic issue (e.g. Wiesepape et al., 2025; Maj et al., 2009) that calls for coordinated and evidence-informed action. Moreover, the lack of cross-national consensus on integrated care models for this population poses a substantial barrier to effective policy development and implementation.

From a methodological standpoint, the use of a Delphi process is appropriate for the stated aim, and the protocol demonstrates several noteworthy strengths. First, the inclusion of people living with SMI, their relatives, caregivers, peer supporters alongside health professionals and decision-makers in the Delphi process is commendable and aligns with WHO recommendations for participatory approaches. Second, the inclusion of six European countries representing diverse healthcare system typologies enhances the potential external validity and transferability of the findings. Third, the questionnaire content is grounded in a prior scoping review and further refined through consortium consultation, strengthening its empirical basis. Finally, adherence to well-established Delphi reporting guidelines (e.g., DelphiStar), along with the clear description of data management procedures, ethical considerations, and study processes, underscores the methodological rigor of the protocol.

Despite these strengths, the manuscript would benefit from revisions to improve transparency and clarity. Having read the manuscript two times, I outline below a number of comments and suggestions for your consideration.

Major issues/Major concerns

1. *Title. Tittle alignment with study aim.* The title refers to “optimal models” of integrated care; however, the Delphi study is designed to achieve consensus on key characteristics rather than to evaluate or compare comprehensive integrated care models. The title would benefit from revision to more accurately reflect the study’s objectives; for example, by referring to “key characteristics” or “priority features” of integrated care models.

2. *Introduction*. The protocol does not specify whether the study focuses on particular types of physical comorbidities (e.g., cardiovascular, metabolic, respiratory) or whether it encompasses all somatic conditions. Given the known heterogeneity in the burden of specific comorbidities among individuals with SMI (Jones et al., 2004), a brief clarification on the intended scope would be helpful for readers.
3. *Methods*. *Sample size adequacy and attrition risk*. The target of 33 participants per country (198 in total) is presented with a general reference to the recommendation of 5–10 experts per stakeholder group. However, the manuscript provides limited discussion on how this sample size was calibrated to account for potential attrition across the three Delphi rounds. In particular, there is limited discussion of how the sample size was calibrated to account for potential attrition across the three Delphi rounds. In particular, differential dropout across stakeholder groups (e.g., individuals living with SMI, who may face greater barriers to sustained participation) could compromise panel representativeness in later rounds. While the authors note that additional participants will be contacted, it would strengthen the protocol to further elaborate on the anticipated impact of high attrition rates and to outline contingency strategies, particularly in cases where dropout is uneven across stakeholder groups.
4. *Methods. Handling of new propositions from the open-ended questions.* The protocol mentions that participants may propose new topics through an open-ended question and that new propositions may be incorporated in Round 2. However, the decision rules for incorporating participant-generated propositions are not fully described. For instance: (1) how will proposals be screened and selected for inclusion? (2) who will make these decisions - all researchers from the EU-MIND consortium or a smaller subgroup? (3) will new propositions undergo content analysis/thematic synthesis prior to inclusion? In the absence of predefined criteria, the inclusion of new items risks introducing bias and reducing procedural transparency. The authors should provide explicit decision rules governing this process.

Minor issues/Minor comments

1. *Introduction — Framing of the EU-MIND consortium.* The manuscript references the European Mental and Physical Health Initiative for People with Severe Mental Disorders (EU-MIND) consortium repeatedly throughout the text as the overarching framework within which this Delphi study is embedded. However, no description of the consortium is provided. The reader is not told what the EU-MIND initiative is, what its overall objectives are, which institutions and countries are involved, how it is governed, or how the present Delphi study fits within its broader programme of work. I would recommend that the authors include a concise paragraph providing a clear description of the EU-MIND consortium. This would greatly help readers contextualize the study and understand its strategic relevance.
2. *Methods – Pilot testing*. The protocol mentions that the questionnaire will be pilot tested by researchers and by individuals with lived experience of SMI. However, no detail is provided on the anticipated number of pilot testers, the criteria for evaluating the pilot outcomes (e.g., clarity, relevance, completion time, accessibility of language), or the types of modifications that could result from this testing. This

section would benefit from further elaboration to allow readers to assess the adequacy of the piloting process.

3. *Methods*. The authors note that participants may belong to more than one stakeholder category. However, the protocol does not describe how this overlap will be handled analytically — specifically, whether participants will be asked to self-select a primary category for subgroup analyses or whether they will be counted in all applicable categories. I would recommend that the authors specify how this will be managed.
4. *Methods*. The protocol states that the decision to proceed to a Delphi third round is contingent upon the attrition rate remaining "within acceptable limits," but these limits are not defined. In the absence of a pre-specified threshold, this decision could be perceived as a post-hoc judgement, potentially raising concerns about the rigour of the process. Specifying a threshold (e.g., retention above a given percentage) would strengthen the protocol's transparency.
5. *Methods*. It is unclear whether the survey includes a "do not know" or "do not wish to answer" response option alongside the 1–9 Likert scale. The expert panel includes stakeholders with very different areas of expertise: people with lived experience may not feel qualified to rate certain governance or funding propositions, while decision-makers may lack direct insight into the daily care experience. The absence of such an option could compel participants to provide ratings on topics outside their area of knowledge, introducing forced responses that do not reflect genuine opinions. This could artificially inflate agreement levels and compromise the validity of the consensus. I would encourage the authors to clarify whether a "do not know" or "prefer not to answer" option is available. If it is, specifying how such responses will be treated analytically would be useful for the reader. If it is not available, I would recommend that the authors justify this design choice and discuss its potential implications for the validity of the results.
6. *Methods*. Figure 1 appears blurred and of low resolution in the submitted manuscript. The authors should provide a higher-quality version of this figure to ensure legibility in the published article. Given the importance of this figure for understanding the overall study process, a clear rendering is essential.
7. *Discussion*. The Discussion mentions that the Delphi results will feed into subsequent EU-MIND phases (health economic evaluation and a feasibility pilot). The authors could strengthen the manuscript by briefly explaining how the consensus findings could be translated into an operational care model specifications, helping readers assess the practical impact potential of the Delphi results.
8. *Discussion/Limitations.* The Discussion section currently mentions sampling and representativeness biases only briefly. However, the Delphi method is associated with a broader range of well-documented biases (see e.g. Sokolov et al. 2025; Bolger et al., 2021) that warrant explicit discussion. These include selection biases, question framing bias, social desirability bias or false consensus effect. I would recommend that the authors expand the limitations section to include a more comprehensive discussion of how these biases could undermine the credibility and interpretability of findings.
