# Supplementary material for: Protocol for a Delphi consensus study to identify priority characteristics of integrated care for individuals with severe mental illness and comorbid physical disorders in Europe
Source: PLoS One. 2026 Jul 9;21(7):e0352089. doi: 10.1371/journal.pone.0352089 (PMC13392973; doi:10.1371/journal.pone.0352089)
Supplement: Attachment — Submitted filename: Point_by_point_response_reviewer_comments_VF.docx [file pone.0352089.s004.docx]

*We thank the editor for the opportunity to revise and resubmit our manuscript entitled ‘Protocol for a Delphi consensus study to identify priority characteristics of integrated care for individuals with severe mental illness and comorbid physical disorders in Europe’. We also thank the reviewer for their time and thoughtful comments. The comments were very clear and concrete, which has made the revision straightforward for us, and the comments have helped us further improve our manuscript in several respects. We have responded to each comment in an itemized fashion and revised our manuscript accordingly. We hope that the revised version of our manuscript is now ready for acceptance and publication in PLOS One. We look forward to hearing from you.*

**Reviewer’s comments**

**Major issues/Major concerns**

1. Title. Tittle alignment with study aim. The title refers to “optimal models” of integrated care; however, the Delphi study is designed to achieve consensus on key characteristics rather than to evaluate or compare comprehensive integrated care models. The title would benefit from revision to more accurately reflect the study’s objectives; for example, by referring to “key characteristics” or “priority features” of integrated care models.

*Response: Thank you for this insightful comment. We fully agree that the original title did not sufficiently reflect the specific objective of the Delphi study, which is to achieve expert consensus on key characteristics rather than to evaluate or compare integrated care models. In response to your suggestion, we have revised the title accordingly to more accurately reflect the study’s aim. The revised title now reads: ‘Protocol for a Delphi consensus study to identify priority characteristics of integrated care for individuals with severe mental illness and comorbid physical disorders in Europe.’ (see page 1, highlighted in yellow).*

2. Introduction. The protocol does not specify whether the study focuses on particular types of physical comorbidities (e.g., cardiovascular, metabolic, respiratory) or whether it encompasses all somatic conditions. Given the known heterogeneity in the burden of specific comorbidities among individuals with SMI (Jones et al., 2004), a brief clarification on the intended scope would be helpful for readers.

*Response: We agree that clarifying the scope of physical comorbidities addressed in the study is important. The EU-MIND research project adopts a global and holistic approach to health and therefore encompasses all types of physical comorbidities, rather than focusing on specific disease categories chosen a priori. This clarification has now been explicitly added to the Introduction, in the section providing further details on the EU-MIND initiative (in response to another of your comments) (see page 5, lines 108-109, highlighted in yellow): ‘Unlike initiatives targeting specific comorbidities, EU‑MIND adopts a comprehensive approach to health encompassing all somatic conditions relevant to individuals with SMI.’.*

3. Methods. Sample size adequacy and attrition risk. The target of 33 participants per country (198 in total) is presented with a general reference to the recommendation of 5-10 experts per stakeholder group. However, the manuscript provides limited discussion on how this sample size was calibrated to account for potential attrition across the three Delphi rounds. In particular, differential dropout across stakeholder groups (e.g., individuals living with SMI, who may face greater barriers to sustained participation) could compromise panel representativeness in later rounds. While the authors note that additional participants will be contacted, it would strengthen the protocol to further elaborate on the anticipated impact of high attrition rates and to outline contingency strategies, particularly in cases where dropout is uneven across stakeholder groups.

*Response: We fully agree that attrition is a key methodological consideration in Delphi studies, particularly when involving heterogeneous stakeholder groups with varying abilities to sustain participation. As noted, individuals living with SMI may face greater barriers to continued engagement, and uneven dropout across groups could compromise panel representativeness in later rounds. In response, we have expanded the Methods section to more explicitly address both the anticipated attrition and the mitigation strategies planned. First, the initial sample size was deliberately set above the commonly recommended threshold of 5-10 experts per stakeholder group, in anticipation of participant loss across successive rounds. Second, specific measures have been implemented to support continued participation by individuals with lived experience of SMI. At first contact, individuals were encouraged to participate throughout the study and were informed that, if they so wished, they could complete the questionnaires with the support of a trusted relative or caregiver. To further minimize attrition for all stakeholder groups, several strategies will be applied: personalized emails addressing each participant by name (sent through the Limesurvey platform), clear and concise questionnaires to avoid participant burden, reminder procedures, and the option to save and return to the survey at any time. In addition, prior to the first questionnaire, we provided a detailed explanation of the Delphi process to all participants to prevent confusion with standard non‑iterative surveys and to reinforce the importance of participation across rounds. Participants will also be kept informed of any adjustments to the data collection timeline to maintain engagement and transparency throughout the study. We have added these points in the manuscript (see page 8, lines 171-179, highlighted in yellow):* ‘*Attrition may disproportionately affect certain stakeholder groups, particularly individuals living with SMI. Specific retention strategies will therefore be implemented. They include a detailed explanation of the iterative nature of the Delphi process prior to the first round, personalized email communications at all stages of the study, concise questionnaires designed to minimize participant burden, systematic reminders procedures, and flexible survey completion options. For participants with SMI, this flexibility includes the possibility of completing questionnaires with the assistance of a trusted relative or caregiver. In addition, adjustments to the study timeline will be proactively communicated to participants to support continued engagement across rounds’.*

4. Methods. Handling of new propositions from the open-ended questions. The protocol mentions that participants may propose new topics through an open-ended question and that new propositions may be incorporated in Round 2. However, the decision rules for incorporating participant-generated propositions are not fully described. For instance: (1) how will proposals be screened and selected for inclusion? (2) who will make these decisions - all researchers from the EU-MIND consortium or a smaller subgroup? (3) will new propositions undergo content analysis/thematic synthesis prior to inclusion? In the absence of predefined criteria, the inclusion of new proposals risks introducing bias and reducing procedural transparency. The authors should provide explicit decision rules governing this process.

*Response: Thank you for this important comment highlighting the need for greater procedural transparency. We agree that explicit decision rules for handling participant‑generated proposals are essential to minimise bias and clarify the methodological process. In response, we have revised the Methods section to more clearly describe how new proposals arising from the open‑ended question will be handled. All additional suggestions will be screened by the coordination team leading the Delphi process. This team will be responsible for reviewing the proposals and deciding on their inclusion based on predefined criteria. Specifically, proposals will be included if they are: (1) relevant to integrated care for individuals with SMI and comorbid physical conditions; (2) sufficiently clear and actionable to be formulated as Delphi items; and (3) not redundant with proposals already included in the questionnaire. Country representatives will be consulted as needed to support accurate interpretation and translation of suggestions submitted in different languages; however, final decisions regarding inclusion will rest with the Delphi coordination team to ensure consistency across countries. New proposals will not undergo a formal content analysis or thematic synthesis between two Delphi rounds; rather, they will be subject to structured screening against the criteria outlined above and, where appropriate, rephrased for clarity while preserving their original meaning. These decision rules have now been explicitly added to the Methods section (see page 12, lines 234-240, highlighted in yellow): ‘They will be included if they meet the following criteria: the topic is relevant to integrated care for individuals with SMI and comorbid physical conditions; they are sufficiently clear and actionable to be operationalized as Delphi items; they are not redundant with proposals already included in the questionnaire. To support accurate interpretation and translation of proposals submitted in different languages, country representatives will be consulted as needed. However, final decisions regarding inclusion will remain the responsibility of the French team, in charge of the Delphi coordination, to ensure methodological consistency across countries’. It should also be noted that, as the open‑ended question invites participants to identify a single essential change they would make to the organisation of care for people with SMI in order to highlight an ultimate priority, we do not anticipate the generation of a large number of new proposals.*

**Minor issues/Minor comments**

1. Introduction — Framing of the EU-MIND consortium. The manuscript references the European Mental and Physical Health Initiative for People with Severe Mental Disorders (EU-MIND) consortium repeatedly throughout the text as the overarching framework within which this Delphi study is embedded. However, no description of the consortium is provided. The reader is not told what the EU-MIND initiative is, what its overall objectives are, which institutions and countries are involved, how it is governed, or how the present Delphi study fits within its broader programme of work. I would recommend that the authors include a concise paragraph providing a clear description of the EU-MIND consortium. This would greatly help readers contextualize the study and understand its strategic relevance.

*Response: Thank you for this valuable suggestion. We agree that providing a more detailed description of the EU‑MIND consortium will help readers better understand the strategic context in which the present Delphi study is embedded. In response, we have added a short paragraph at the end of the Introduction summarizing in more details the initiative (see pages 5-6, lines 103-116, highlighted in yellow): ‘It is conducted as part of the European Mental and Physical Health Initiative for People with Severe Mental Disorders (EU-MIND, https://eumind.eu/), funded by the 2024 European Partnership on Transforming Health and Care Systems. EU‑MIND is a three‑year interdisciplinary consortium involving Denmark, Finland, France, Italy, Poland, and Sweden. Its aim is to develop a European solution to improve the organization of care for people with SMI and co‑occurring physical disorders. Unlike initiatives targeting specific comorbidities, EU‑MIND adopts a comprehensive approach to health encompassing all somatic conditions relevant to individuals with SMI. The project is structured into several work packages covering: (1) analysis of nationwide health claims databases to identify gaps in care pathways and mortality outcomes; (2) an umbrella review of existing integrated care programs for people with SMI; (3) the Delphi consensus process reported in this article, focused on identifying priority characteristics of integrated care to support; (4) a health‑economic evaluation of societal attitudes toward implementing these priority features and its financial impact; and (5) a feasibility pilot of such implementation in real‑world settings. A dedicated dissemination work package also supports knowledge translation across Europe’. Furthermore, the subsequent steps of the Delphi method were described in the discussion section (see page 16, lines 337-340): ‘These phases will include a health economic evaluation, assessing societal knowledge, attitudes, practices, willingness to pay, and the financial implications of implementing this consensus‑based integrated care model for people with SMI, as well as a feasibility pilot study examining the model’s practical applicability and sustainability.’.*

2. Methods – Pilot testing. The protocol mentions that the questionnaire will be pilot tested by researchers and by individuals with lived experience of SMI. However, no detail is provided on the anticipated number of pilot testers, the criteria for evaluating the pilot outcomes (e.g., clarity, relevance, completion time, accessibility of language), or the types of modifications that could result from this testing. This section would benefit from further elaboration to allow readers to assess the adequacy of the piloting process.

*Response: We agree that additional detail regarding the pilot testing process is necessary to allow readers to assess its adequacy and rigor. We have therefore expanded the Methods section to clarify both the composition of the pilot testing sample and the criteria used to evaluate the questionnaire. The questionnaire will be pilot tested with three additional complementary groups: individuals with lived experience of SMI, lay members of the general public with no prior involvement in the project, and informed experts external to the research team. Pilot testing will assess completion time, overall clarity of the study aims, and the wording and internal coherence of questionnaire proposals, with particular attention to accessibility of language. Feedback from this process will be used to revise the questionnaire prior to its implementation in the Delphi rounds. These precisions have been added to the manuscript (page 11, lines 217-222, highlighted in yellow): ‘The questionnaire will also be pilot tested by individuals with lived experience of SMI, lay members of the general population with no prior involvement in the project, and informed experts external to the research team. Pilot testing will assess completion time, the overall clarity of the study aims, and the wording and internal coherence of questionnaire proposals, with particular attention to accessibility of language. Feedback from this process will be used to revise the questionnaire prior to its implementation in the Delphi rounds.’.* *The results article will detail the final numbers of pilot testers by category and acknowledge their participation.*

3. Methods. The authors note that participants may belong to more than one stakeholder category. However, the protocol does not describe how this overlap will be handled analytically – specifically, whether participants will be asked to self-select a primary category for subgroup analyses or whether they will be counted in all applicable categories. I would recommend that the authors specify how this will be managed.

*Response: We agree that the management of overlapping stakeholder categories should be clearly specified. In this study, participants who belong to more than one stakeholder group will be included in all applicable categories for descriptive and subgroup analyses, rather than being asked to select a single primary category. This approach reflects the reality of multiple professional and experiential roles and avoids arbitrarily prioritising one form of expertise over another. We have now clarified this analytical strategy in the Methods section (see page 14, lines 286-288, highlighted in yellow): ‘Participants will be included in all categories to which they report belonging, and analyses by expert category will therefore be conducted using overlapping groups. The number of participants contributing to each category will be reported accordingly.’.*

4. Methods. The protocol states that the decision to proceed to a Delphi third round is contingent upon the attrition rate remaining ‘within acceptable limits,’ but these limits are not defined. In the absence of a pre-specified threshold, this decision could be perceived as a post-hoc judgement, potentially raising concerns about the rigour of the process. Specifying a threshold (e.g., retention above a given percentage) would strengthen the protocol’s transparency.

*Response: Thank you for this important comment. We agree that the criteria governing the decision to proceed to a third Delphi round must be explicitly pre‑specified to avoid any perception of post‑hoc judgement. We have therefore revised the protocol to define clear, a priori stopping and continuation criteria. While a minimum of two rounds is inherent to the Delphi methodology, the decision to conduct an additional round must balance the potential informational gain against the risk of excessive attrition and artificial consensus emerging from participant fatigue. To address this, and in line with methodological recommendations, we have now specified both an attrition threshold and content‑based criteria to guide this decision in a transparent and reproducible manner. Specifically, we have defined an attrition rate exceeding 40% as a stopping criterion after the second round. While the Delphi literature does not prescribe a universally accepted numerical threshold, previous methodological work highlights that high attrition rates (often discussed as exceeding approximately 20–30%) can compromise the validity and interpretability of findings. The threshold that we chose is therefore conservative relative to commonly cited recommendations but was deemed appropriate given the unusually large and heterogeneous initial panel (nearly 200 participants), in which some degree of attrition is expected. In addition to attrition, the decision to proceed to a third round will be informed by the stability of ratings across rounds and the proportion of proposals that have not yet reached consensus. Consistent with methodological guidance on Delphi studies, stability will be examined at multiple levels, including overall panel stability and stability within relevant subgroups (e.g., by country or stakeholder category) (Khodyakov, D., Grant, S., Kroger, J., Bauman, M., 2023. RAND Methodological Guidance for Conducting and Critically Appraising Delphi Panels. RAND Corporation). Individual‑level changes in ratings across rounds will also be explored to identify persistent variability. If meaningful instability remains while attrition stays below the predefined threshold, a third round will be undertaken, provided that a substantial proportion of proposals (≥25%) have not yet reached consensus. These criteria have now been explicitly incorporated into the Methods section (see page 13, lines 268-272, highlighted in yellow): ‘A third Delphi round will be conducted only if attrition remains below 40%, rating stability analyses indicate residual instability at the panel, individual or subgroup level (i.e., by country or stakeholder category) (47), and at least 25% of proposals have not reached consensus after the second round. The decision to proceed will therefore be based on the a priori assessment that an additional round is likely to generate robust and meaningful additional data to inform the final interpretation of the consensus.’.*

5. Methods. It is unclear whether the survey includes a ‘do not know’ or ‘do not wish to answer’ response option alongside the 1-9 Likert scale. The expert panel includes stakeholders with very different areas of expertise: people with lived experience may not feel qualified to rate certain governance or funding propositions, while decision-makers may lack direct insight into the daily care experience. The absence of such an option could compel participants to provide ratings on topics outside their area of knowledge, introducing forced responses that do not reflect genuine opinions. This could artificially inflate agreement levels and compromise the validity of the consensus. I would encourage the authors to clarify whether a ‘do not know’ or ‘prefer not to answer’ option is available. If it is, specifying how such responses will be treated analytically would be useful for the reader. If it is not available, I would recommend that the authors justify this design choice and discuss its potential implications for the validity of the results.

*Response: Thank you for raising this important point. We agree that it is best practice in survey design to offer respondents the option to refrain from answering or to select ‘I don’t know’. However, such options are not always included in Delphi studies, as participants are specifically recruited for their expertise in the subject matter. While the questionnaire will not include a separate ‘do not know’ or ‘prefer not to answer’ response option, participants will be explicitly instructed to use a score of 5 (midpoint) to indicate uncertainty or insufficient knowledge regarding a given proposal. The survey instructions will state: ‘If you are unsure about a proposal, you may select 5 and move on to the next question; this response will be interpreted as ‘do not know’. You will have the opportunity to re‑evaluate certain proposals in subsequent rounds.’. From an analytical perspective, midpoint ratings will not be interpreted as either agreement or disagreement and will therefore be excluded from the calculation of consensus thresholds, as would have a ’do not know’ option. This approach prevents forced responses from contributing to consensus and it was chosen to maintain a consistent rating scale while ensuring accessibility for participants with diverse backgrounds and areas of expertise. We have clarified this in the Methods section (see page 9, lines 195-198, highlighted in yellow): ‘Despite the absence of an explicit ‘do not know’ or ‘prefer not to answer’ response option, participants will be instructed to use the midpoint value of the scale (score = 5) to indicate uncertainty or insufficient knowledge regarding a given proposal.’.*

6. Methods. Figure 1 appears blurred and of low resolution in the submitted manuscript. The authors should provide a higher-quality version of this figure to ensure legibility in the published article. Given the importance of this figure for understanding the overall study process, a clear rendering is essential.

*Response: Thank you for drawing our attention to this issue. We agree that Figure 1 plays a key role in conveying the overall study process. A higher‑resolution version of the figure has now been provided to improve readability and visual clarity, and the figure has been updated in the revised manuscript (see page 14).*

7. Discussion. The Discussion mentions that the Delphi results will feed into subsequent EU-MIND phases (health economic evaluation and a feasibility pilot). The authors could strengthen the manuscript by briefly explaining how the consensus findings could be translated into operational care model specifications, helping readers assess the practical impact potential of the Delphi results.

*Response: We agree that the original Discussion did not sufficiently clarify how the Delphi consensus findings will be translated into operational specifications of key characteristics of an integrated care model to support and further study in subsequent steps of the EU-MIND research project. Specifically, the proposals reaching consensus will be mapped onto key dimensions of integrated care for multimorbidity (e.g. governance, workforce, service delivery…) and translated into actionable design elements. These elements will constitute the formal specification of the integrated care model that will then be used as the foundation for the subsequent health economic evaluation and the feasibility pilot of the EU-MIND research project. This clarification has been added to the Discussion section (page 16, lines 333-337, highlighted in yellow): ‘The proposals reaching consensus will be mapped onto key dimensions of integrated care for multimorbidity (e.g. governance, workforce, service delivery…) and translated into actionable design elements. Together, these elements will constitute the formal specification of the integrated care model which will serve as the basis for the subsequent phases of the EU‑MIND project.’.*

8. Discussion/Limitations. The Discussion section currently mentions sampling and representativeness biases only briefly. However, the Delphi method is associated with a broader range of well-documented biases (see e.g. Sokolov et al. 2025; Bolger et al., 2021) that warrant explicit discussion. These include selection biases, question framing bias, social desirability bias or false consensus effect. I would recommend that the authors expand the limitations section to include a more comprehensive discussion of how these biases could undermine the credibility and interpretability of findings.”.

*Response: Thank you for this insightful comment. We agree that the initial Discussion did not sufficiently reflect the range of methodological biases documented in the literature. We have therefore expanded the Limitations section to explicitly acknowledge these potential biases. In addition, we describe the procedural choices adopted to mitigate their impact where possible (e.g. controlled feedback, anonymity, inclusion of diverse stakeholder groups), while recognising that these risks cannot be entirely eliminated. This expanded discussion aims to provide a more balanced and transparent assessment of the strengths and limitations of the Delphi process used (pages 16-17, lines 347-360, highlighted in yellow): ‘Nevertheless, the results of this study will have to be interpreted in light of the inherent limitations of the Delphi method (51). Selection bias may arise from reliance on voluntary participation and from the composition of the expert panel, as participation is influenced by availability, motivation, and prior interest in the topic, potentially leading to the over‑representation of certain viewpoints. In addition, the framing and wording of questionnaire proposals may shape participants’ interpretations and responses, despite iterative refinement and pilot testing. Although anonymity and controlled feedback are intended to reduce domination effects, social desirability bias and normative pressures may still operate, particularly across rounds, as participants are exposed to aggregated group responses. Such dynamics may contribute to convergence that reflects accommodation or compliance rather than genuine changes in individual opinion. Attrition across rounds may further accentuate these effects if remaining participants hold relatively homogeneous views. While several design features of our Delphi survey were implemented to mitigate these risks, including stakeholder diversity, anonymized rating procedures, and pre‑specified stopping criteria, these biases cannot be fully eliminated and will have to be considered when interpreting the findings.’.*
