# Supplementary material for: Protocol for a Delphi consensus study to identify priority characteristics of integrated care for individuals with severe mental illness and comorbid physical disorders in Europe
Source: PLoS One. 2026 Jul 9;21(7):e0352089. doi: 10.1371/journal.pone.0352089 (PMC13392973; doi:10.1371/journal.pone.0352089)
Supplement: Peer review history

# Peer review: Decision Letter 0

**DOI:** 10.1371/journal.pone.0352089.r001

**Authors:** Eleni Petkari

7 Apr 2026

PONE-D-26-08246

Protocol for a Delphi consensus study to identify optimal models of integrated care for individuals with severe mental illness and comorbid physical disorders in Europe

PLOS One

Dear Dr. touitou-burckard,

Thank you for submitting your manuscript to PLOS ONE. After careful consideration, we feel that it has merit but does not fully meet PLOS ONE’s publication criteria as it currently stands. Therefore, we invite you to submit a revised version of the manuscript that addresses the points raised during the review process.

Please pay careful attention to the Reviewers' comment regarding sampling-selection bias.

Please submit your revised manuscript by May 22 2026 11:59PM. If you will need more time than this to complete your revisions, please reply to this message or contact the journal office at plosone@plos.org. When you're ready to submit your revision, log on to https://www.editorialmanager.com/pone/ and select the 'Submissions Needing Revision' folder to locate your manuscript file.

Please include the following items when submitting your revised manuscript:

- A letter that responds to each point raised by the academic editor and reviewer(s). You should upload this letter as a separate file labeled 'Response to Reviewers'.
- A marked-up copy of your manuscript that highlights changes made to the original version. You should upload this as a separate file labeled 'Revised Manuscript with Track Changes'.
- An unmarked version of your revised paper without tracked changes. You should upload this as a separate file labeled 'Manuscript'.

If you would like to make changes to your financial disclosure, please include your updated statement in your cover letter. Guidelines for resubmitting your figure files are available below the reviewer comments at the end of this letter.

If applicable, we recommend that you deposit your laboratory protocols in protocols.io to enhance the reproducibility of your results. Protocols.io assigns your protocol its own identifier (DOI) so that it can be cited independently in the future. For instructions see: https://journals.plos.org/plosone/s/submission-guidelines#loc-laboratory-protocols. Additionally, PLOS ONE offers an option for publishing peer-reviewed Lab Protocol articles, which describe protocols hosted on protocols.io. Read more information on sharing protocols at https://plos.org/protocols?utm_medium=editorial-email&utm_source=authorletters&utm_campaign=protocols.

As the corresponding author, your ORCID iD is verified in the submission system and will appear in the published article. PLOS supports the use of ORCID, and we encourage all coauthors to register for an ORCID iD and use it as well. Please encourage your coauthors to verify their ORCID iD within the submission system before final acceptance, as unverified ORCID iDs will not appear in the published article. *Only*  the individual author can complete the verification step; PLOS staff *cannot*  verify ORCID iDs on behalf of authors.

We look forward to receiving your revised manuscript.

Kind regards,

Eleni Petkari

Academic Editor

PLOS One

Journal Requirements:

When submitting your revision, we need you to address these additional requirements.

1. Please ensure that your manuscript meets PLOS ONE's style requirements, including those for file naming. The PLOS ONE style templates can be found at

https://journals.plos.org/plosone/s/file?id=wjVg/PLOSOne_formatting_sample_main_body.pdf and

https://journals.plos.org/plosone/s/file?id=ba62/PLOSOne_formatting_sample_title_authors_affiliations.pdf

2. Please include a complete copy of PLOS’ questionnaire on inclusivity in global research in your revised manuscript. Our policy for research in this area aims to improve transparency in the reporting of research performed outside of researchers’ own country or community. The policy applies to researchers who have travelled to a different country to conduct research, research with Indigenous populations or their lands, and research on cultural artefacts. The questionnaire can also be requested at the journal’s discretion for any other submissions, even if these conditions are not met.  Please find more information on the policy and a link to download a blank copy of the questionnaire here: https://journals.plos.org/plosone/s/best-practices-in-research-reporting. Please upload a completed version of your questionnaire as Supporting Information when you resubmit your manuscript.

3. We note that the grant information you provided in the ‘Funding Information’ and ‘Financial Disclosure’ sections do not match.

When you resubmit, please ensure that you provide the correct grant numbers for the awards you received for your study in the ‘Funding Information’ section.

4. When completing the data availability statement of the submission form, you indicated that you will make your data available on acceptance. We strongly recommend all authors decide on a data sharing plan before acceptance, as the process can be lengthy and hold up publication timelines. Please note that, though access restrictions are acceptable now, your entire data will need to be made freely accessible if your manuscript is accepted for publication. This policy applies to all data except where public deposition would breach compliance with the protocol approved by your research ethics board. If you are unable to adhere to our open data policy, please kindly revise your statement to explain your reasoning and we will seek the editor's input on an exemption. Please be assured that, once you have provided your new statement, the assessment of your exemption will not hold up the peer review process.

5. One of the noted authors is a group or consortium EU-MIND Consortium Collaborators. In addition to naming the author group, please list the individual authors and affiliations within this group in the acknowledgments section of your manuscript. Please also indicate clearly a lead author for this group along with a contact email address.

6. If the reviewer comments include a recommendation to cite specific previously published works, please review and evaluate these publications to determine whether they are relevant and should be cited. There is no requirement to cite these works unless the editor has indicated otherwise.

[Note: HTML markup is below. Please do not edit.]

Reviewers' comments:

Reviewer's Responses to Questions

**Comments to the Author**

1. Does the manuscript provide a valid rationale for the proposed study, with clearly identified and justified research questions?

The research question outlined is expected to address a valid academic problem or topic and contribute to the base of knowledge in the field.

Reviewer #1: Yes

Reviewer #2: Yes

**********

2. Is the protocol technically sound and planned in a manner that will lead to a meaningful outcome and allow testing the stated hypotheses?

The manuscript should describe the methods in sufficient detail to prevent undisclosed flexibility in the experimental procedure or analysis pipeline, including sufficient outcome-neutral conditions (e.g. necessary controls, absence of floor or ceiling effects) to test the proposed hypotheses and a statistical power analysis where applicable. As there may be aspects of the methodology and analysis which can only be refined once the work is undertaken, authors should outline potential assumptions and explicitly describe what aspects of the proposed analyses, if any, are exploratory.

Reviewer #1: Yes

Reviewer #2: Yes

**********

3. Is the methodology feasible and described in sufficient detail to allow the work to be replicable?

Descriptions of methods and materials in the protocol should be reported in sufficient detail for another researcher to reproduce all experiments and analyses. The protocol should describe the appropriate controls, sample size calculations, and replication needed to ensure that the data are robust and reproducible.

Reviewer #1: Yes

Reviewer #2: Yes

**********

4. Have the authors described where all data underlying the findings will be made available when the study is complete?

The PLOS Data policy requires authors to make all data underlying the findings described in their manuscript fully available without restriction, with rare exception, at the time of publication. The data should be provided as part of the manuscript or its supporting information, or deposited to a public repository. For example, in addition to summary statistics, the data points behind means, medians and variance measures should be available. If there are restrictions on publicly sharing data—e.g. participant privacy or use of data from a third party—those must be specified.

Reviewer #1: Yes

Reviewer #2: Yes

**********

5. Is the manuscript presented in an intelligible fashion and written in standard English?

PLOS ONE does not copyedit accepted manuscripts, so the language in submitted articles must be clear, correct, and unambiguous. Any typographical or grammatical errors should be corrected at revision, so please note any specific errors here.

Reviewer #1: Yes

Reviewer #2: Yes

**********

6. Review Comments to the Author

Please use the space provided to explain your answers to the questions above and, if applicable, provide comments about issues authors must address before this protocol can be accepted for publication. You may also include additional comments for the author, including concerns about research or publication ethics.

You may also provide optional suggestions and comments to authors that they might find helpful in planning their study.

(Please upload your review as an attachment if it exceeds 20,000 characters)

Reviewer #1: Review Comments to the Author uploaded as an attachment.

Confidential Comments to the Editor uploaded as an attachment.

Reviewer #2: The protocol is well‑structured, methodologically grounded, and aligned with established Delphi guidelines. It addresses an important gap in the organization of integrated care for people with severe mental illness (SMI).

The protocol shows several notable strengths. It provides a clear and well‑founded rationale for using the Delphi method in a context where robust evidence is scarce. The inclusion of a multinational, multidisciplinary panel enhances the relevance and transferability of the results. The study also adheres to established methodological and ethical standards, and the translation process is carefully designed, combining machine translation with bilingual human review to ensure cultural and linguistic accuracy.

As a minor point for clarification, in the Sampling and participants section, in the sentence “To ensure the heterogeneity of the expert panel, which is essential to the quality and relevance of the consensus reached (43), several subcategories within each stakeholder group will be considered (Table 1).” it would be helpful to add a brief statement indicating what the reader can expect to find in Table 1. Specifically, you could mention that the table outlines the planned number of participants in each stakeholder category, which is intended to ensure adequate representativeness of the sample.

A more substantive concern relates to the potential for selection bias in the recruitment of participants for the Delphi process. As the protocol indicates that “experts are recruited through the networks of national research teams”, this approach may inadvertently limit the diversity of perspectives included and introduce selection bias. Relying primarily on existing professional networks could restrict the representativeness of the expert panel. To mitigate this risk and strengthen the methodological robustness of the study, we encourage the authors to consider complementary and more inclusive recruitment strategies, such as open calls or collaboration with patient and family associations, which could broaden the sampling frame and enhance the diversity and representativeness of the panel.

Another important methodological consideration concerns the flexibility of the Delphi process to accommodate emergent issues. Recent methodological guidance emphasizes that a key strength of the Delphi technique lies in its iterative and adaptive nature, allowing new themes or items proposed by participants to be introduced and refined across rounds, particularly in the early phases of the process (Diamond et al., 2014; Niederberger & Spranger, 2020). The authors note that the second round is intended to allow for further reflection and potential convergence of opinions, and that it may incorporate new propositions suggested by participants. However, it remains unclear whether and how these newly introduced propositions would be systematically incorporated into the analysis framework. We believe that, throughout the Delphi rounds, participants may identify topics or dimensions not initially anticipated by the authors but still considered relevant by the panel. We therefore encourage the authors to further clarify the procedures for capturing, reviewing, and analytically integrating such emergent themes. Explicitly addressing this point may help avoid excessive protocol rigidity, ensure that participants perspectives are fully reflected, and ultimately enhance the completeness, credibility, and validity of the resulting consensus.

**********

7. PLOS authors have the option to publish the peer review history of their article (what does this mean?). If published, this will include your full peer review and any attached files.

If you choose “no”, your identity will remain anonymous but your review may still be made public.

**Do you want your identity to be public for this peer review?**  For information about this choice, including consent withdrawal, please see our Privacy Policy.

Reviewer #1: No

Reviewer #2: **Yes:** Quintí Foguet-Boreu

**********

[NOTE: If reviewer comments were submitted as an attachment file, they will be attached to this email and accessible via the submission site. Please log into your account, locate the manuscript record, and check for the action link "View Attachments". If this link does not appear, there are no attachment files.]

To ensure your figures meet our technical requirements, please review our figure guidelines: https://journals.plos.org/plosone/s/figures

You may also use PLOS’s free figure tool, NAAS, to help you prepare publication quality figures: https://journals.plos.org/plosone/s/figures#loc-tools-for-figure-preparation.

NAAS will assess whether your figures meet our technical requirements by comparing each figure against our figure specifications.

Attachment Submitted filename: Review manuscript PONE-D-26-08246_Comments to Authors.pdf

# Author comment: Author response to Decision Letter 1

**DOI:** 10.1371/journal.pone.0352089.r002

1 Jun 2026

We thank the editor for the opportunity to revise and resubmit our manuscript entitled ‘Protocol for a Delphi consensus study to identify priority characteristics of integrated care for individuals with severe mental illness and comorbid physical disorders in Europe’. We also thank the reviewer for their time and thoughtful comments. The comments were very clear and concrete, which has made the revision straightforward for us, and the comments have helped us further improve our manuscript in several respects. We have responded to each comment in an itemized fashion and revised our manuscript accordingly. We hope that the revised version of our manuscript is now ready for acceptance and publication in PLOS One. We look forward to hearing from you.

Reviewer’s comments

Major issues/Major concerns

1. Title. Tittle alignment with study aim. The title refers to “optimal models” of integrated care; however, the Delphi study is designed to achieve consensus on key characteristics rather than to evaluate or compare comprehensive integrated care models. The title would benefit from revision to more accurately reflect the study’s objectives; for example, by referring to “key characteristics” or “priority features” of integrated care models.

Response: Thank you for this insightful comment. We fully agree that the original title did not sufficiently reflect the specific objective of the Delphi study, which is to achieve expert consensus on key characteristics rather than to evaluate or compare integrated care models. In response to your suggestion, we have revised the title accordingly to more accurately reflect the study’s aim. The revised title now reads: ‘Protocol for a Delphi consensus study to identify priority characteristics of integrated care for individuals with severe mental illness and comorbid physical disorders in Europe.’ (see page 1, highlighted in yellow).

2. Introduction. The protocol does not specify whether the study focuses on particular types of physical comorbidities (e.g., cardiovascular, metabolic, respiratory) or whether it encompasses all somatic conditions. Given the known heterogeneity in the burden of specific comorbidities among individuals with SMI (Jones et al., 2004), a brief clarification on the intended scope would be helpful for readers.

Response: We agree that clarifying the scope of physical comorbidities addressed in the study is important. The EU-MIND research project adopts a global and holistic approach to health and therefore encompasses all types of physical comorbidities, rather than focusing on specific disease categories chosen a priori. This clarification has now been explicitly added to the Introduction, in the section providing further details on the EU-MIND initiative (in response to another of your comments) (see page 5, lines 108-109, highlighted in yellow): ‘Unlike initiatives targeting specific comorbidities, EU‑MIND adopts a comprehensive approach to health encompassing all somatic conditions relevant to individuals with SMI.’.

3. Methods. Sample size adequacy and attrition risk. The target of 33 participants per country (198 in total) is presented with a general reference to the recommendation of 5-10 experts per stakeholder group. However, the manuscript provides limited discussion on how this sample size was calibrated to account for potential attrition across the three Delphi rounds. In particular, differential dropout across stakeholder groups (e.g., individuals living with SMI, who may face greater barriers to sustained participation) could compromise panel representativeness in later rounds. While the authors note that additional participants will be contacted, it would strengthen the protocol to further elaborate on the anticipated impact of high attrition rates and to outline contingency strategies, particularly in cases where dropout is uneven across stakeholder groups.

Response: We fully agree that attrition is a key methodological consideration in Delphi studies, particularly when involving heterogeneous stakeholder groups with varying abilities to sustain participation. As noted, individuals living with SMI may face greater barriers to continued engagement, and uneven dropout across groups could compromise panel representativeness in later rounds. In response, we have expanded the Methods section to more explicitly address both the anticipated attrition and the mitigation strategies planned. First, the initial sample size was deliberately set above the commonly recommended threshold of 5-10 experts per stakeholder group, in anticipation of participant loss across successive rounds. Second, specific measures have been implemented to support continued participation by individuals with lived experience of SMI. At first contact, individuals were encouraged to participate throughout the study and were informed that, if they so wished, they could complete the questionnaires with the support of a trusted relative or caregiver. To further minimize attrition for all stakeholder groups, several strategies will be applied: personalized emails addressing each participant by name (sent through the Limesurvey platform), clear and concise questionnaires to avoid participant burden, reminder procedures, and the option to save and return to the survey at any time. In addition, prior to the first questionnaire, we provided a detailed explanation of the Delphi process to all participants to prevent confusion with standard non‑iterative surveys and to reinforce the importance of participation across rounds. Participants will also be kept informed of any adjustments to the data collection timeline to maintain engagement and transparency throughout the study. We have added these points in the manuscript (see page 8, lines 171-179, highlighted in yellow): ‘Attrition may disproportionately affect certain stakeholder groups, particularly individuals living with SMI. Specific retention strategies will therefore be implemented. They include a detailed explanation of the iterative nature of the Delphi process prior to the first round, personalized email communications at all stages of the study, concise questionnaires designed to minimize participant burden, systematic reminders procedures, and flexible survey completion options. For participants with SMI, this flexibility includes the possibility of completing questionnaires with the assistance of a trusted relative or caregiver. In addition, adjustments to the study timeline will be proactively communicated to participants to support continued engagement across rounds’.

4. Methods. Handling of new propositions from the open-ended questions. The protocol mentions that participants may propose new topics through an open-ended question and that new propositions may be incorporated in Round 2. However, the decision rules for incorporating participant-generated propositions are not fully described. For instance: (1) how will proposals be screened and selected for inclusion? (2) who will make these decisions - all researchers from the EU-MIND consortium or a smaller subgroup? (3) will new propositions undergo content analysis/thematic synthesis prior to inclusion? In the absence of predefined criteria, the inclusion of new proposals risks introducing bias and reducing procedural transparency. The authors should provide explicit decision rules governing this process.

Response: Thank you for this important comment highlighting the need for greater procedural transparency. We agree that explicit decision rules for handling participant‑generated proposals are essential to minimise bias and clarify the methodological process. In response, we have revised the Methods section to more clearly describe how new proposals arising from the open‑ended question will be handled. All additional suggestions will be screened by the coordination team leading the Delphi process. This team will be responsible for reviewing the proposals and deciding on their inclusion based on predefined criteria. Specifically, proposals will be included if they are: (1) relevant to integrated care for individuals with SMI and comorbid physical conditions; (2) sufficiently clear and actionable to be formulated as Delphi items; and (3) not redundant with proposals already included in the questionnaire. Country representatives will be consulted as needed to support accurate interpretation and translation of suggestions submitted in different languages; however, final decisions regarding inclusion will rest with the Delphi coordination team to ensure consistency across countries. New proposals will not undergo a formal content analysis or thematic synthesis between two Delphi rounds; rather, they will be subject to structured screening against the criteria outlined above and, where appropriate, rephrased for clarity while preserving their original meaning. These decision rules have now been explicitly added to the Methods section (see page 12, lines 234-240, highlighted in yellow): ‘They will be included if they meet the following criteria: the topic is relevant to integrated care for individuals with SMI and comorbid physical conditions; they are sufficiently clear and actionable to be operationalized as Delphi items; they are not redundant with proposals already included in the questionnaire. To support accurate interpretation and translation of proposals submitted in different languages, country representatives will be consulted as needed. However, final decisions regarding inclusion will remain the responsibility of the French team, in charge of the Delphi coordination, to ensure methodological consistency across countries’. It should also be noted that, as the open‑ended question invites participants to identify a single essential change they would make to the organisation of care for people with SMI in order to highlight an ultimate priority, we do not anticipate the generation of a large number of new proposals.

Minor issues/Minor comments

1. Introduction — Framing of the EU-MIND consortium. The manuscript references the European Mental and Physical Health Initiative for People with Severe Mental Disorders (EU-MIND) consortium repeatedly throughout the text as the overarching framework within which this Delphi study is embedded. However, no description of the consortium is provided. The reader is not told what the EU-MIND initiative is, what its overall objectives are, which institutions and countries are involved, how it is governed, or how the present Delphi study fits within its broader programme of work. I would recommend that the authors include a concise paragraph providing a clear description of the EU-MIND consortium. This would greatly help readers contextualize the study and understand its strategic relevance.

Response: Thank you for this valuable suggestion. We agree that providing a more detailed description of the EU‑MIND consortium will help readers better understand the strategic context in which the present Delphi study is embedded. In response, we have added a short paragraph at the end of the Introduction summarizing in more details the initiative (see pages 5-6, lines 103-116, highlighted in yellow): ‘It is conducted as part of the European Mental and Physical Health Initiative for People with Severe Mental Disorders (EU-MIND, https://eumind.eu/), funded by the 2024 European Partnership on Transforming Health and Care Systems. EU‑MIND is a three‑year interdisciplinary consortium involving Denmark, Finland, France, Italy, Poland, and Sweden. Its aim is to develop a European solution to improve the organization of care for people with SMI and co‑occurring physical disorders. Unlike initiatives targeting specific comorbidities, EU‑MIND adopts a comprehensive approach to health encompassing all somatic conditions relevant to individuals with SMI. The project is structured into several work packages covering: (1) analysis of nationwide health claims databases to identify gaps in care pathways and mortality outcomes; (2) an umbrella review of existing integrated care programs for people with SMI; (3) the Delphi consensus process reported in this article, focused on identifying priority characteristics of integrated care to support; (4) a health‑economic evaluation of societal attitudes toward implementing these priority features and its financial impact; and (5) a feasibility pilot of such implementation in real‑world settings. A dedicated dissemination work package also supports knowledge translation across Europe’. Furthermore, the subsequent steps of the Delphi method were described in the discussion section (see page 16, lines 337-340): ‘These phases will include a health economic evaluation, assessing societal knowledge, attitudes, practices, willingness to pay, and the financial implications of implementing this consensus‑based integrated care model for people with SMI, as well as a feasibility pilot study examining the model’s practical applicability and sustainability.’.

2. Methods – Pilot testing. The protocol mentions that the questionnaire will be pilot tested by researchers and by individuals with lived experience of SMI. However, no detail is provided on the anticipated number of pilot testers, the criteria for evaluating the pilot outcomes (e.g., clarity, relevance, completion time, accessibility of language), or the types of modifications that could result from this testing. This section would benefit from further elaboration to allow readers to assess the adequacy of the piloting process.

Response: We agree that additional detail regarding the pilot testing process is necessary to allow readers to assess its adequacy and rigor. We have therefore expanded the Methods section to clarify both the composition of the pilot testing sample and the criteria used to evaluate the questionnaire. The questionnaire will be pilot tested with three additional complementary groups: individuals with lived experience of SMI, lay members of the general public with no prior involvement in the project, and informed experts external to the research team. Pilot testing will assess completion time, overall clarity of the study aims, and the wording and internal coherence of questionnaire proposals, with particular attention to accessibility of language. Feedback from this process will be used to revise the questionnaire prior to its implementation in the Delphi rounds. These precisions have been added to the manuscript (page 11, lines 217-222, highlighted in yellow): ‘The questionnaire will also be pilot tested by individuals with lived experience of SMI, lay members of the general population with no prior involvement in the project, and informed experts external to the research team. Pilot testing will assess completion time, the overall clarity of the study aims, and the wording and internal coherence of questionnaire proposals, with particular attention to accessibility of language. Feedback from this process will be used to revise the questionnaire prior to its implementation in the Delphi rounds.’. The results article will detail the final numbers of pilot testers by category and acknowledge their participation.

3. Methods. The authors note that participants may belong to more than one stakeholder category. However, the protocol does not describe how this overlap will be handled analytically – specifically, whether participants will be asked to self-select a primary category for subgroup analyses or whether they will be counted in all applicable categories. I would recommend that the authors specify how this will be managed.

Response: We agree that the management of overlapping stakeholder categories should be clearly specified. In this study, participants who belong to more than one stakeholder group will be included in all applicable categories for descriptive and subgroup analyses, rather than being asked to select a single primary category. This approach reflects the reality of multiple professional and experiential roles and avoids arbitrarily prioritising one form of expertise over another. We have now clarified this analytical strategy in the Methods section (see page 14, lines 286-288, highlighted in yellow): ‘Participants will be included in all categories to which they report belonging, and analyses by expert category will therefore be conducted using overlapping groups. The number of par

Attachment Submitted filename: Point_by_point_response_reviewer_comments_VF.docx

# Editor report: Decision Letter 1

**DOI:** 10.1371/journal.pone.0352089.r003

**Authors:** Eleni Petkari, Eleni Petkari

4 Jun 2026

Protocol for a Delphi consensus study to identify priority characteristics of integrated care for individuals with severe mental illness and comorbid physical disorders in Europe

PONE-D-26-08246R1

Dear Dr. touitou-burckard,

We’re pleased to inform you that your manuscript has been judged scientifically suitable for publication and will be formally accepted for publication once it meets all outstanding technical requirements.

Within one week, you’ll receive an e-mail detailing the required amendments. When these have been addressed, you’ll receive a formal acceptance letter and your manuscript will be scheduled for publication.

An invoice will be generated when your article is formally accepted. Please note, if your institution has a publishing partnership with PLOS and your article meets the relevant criteria, all or part of your publication costs will be covered. Please make sure your user information is up-to-date by logging into Editorial Manager at Editorial Manager® and clicking the ‘Update My Information' link at the top of the page. For questions related to billing, please contact billing support.

If your institution or institutions have a press office, please notify them about your upcoming paper to help maximize its impact. If they’ll be preparing press materials, please inform our press team as soon as possible -- no later than 48 hours after receiving the formal acceptance. Your manuscript will remain under strict press embargo until 2 pm Eastern Time on the date of publication. For more information, please contact onepress@plos.org.

Kind regards,

Eleni Petkari

Academic Editor

PLOS One

Additional Editor Comments (optional):

Reviewers' comments:

# Editor report: Acceptance letter

**DOI:** 10.1371/journal.pone.0352089.r004

**Authors:** Eleni Petkari, Eleni Petkari

PONE-D-26-08246R1

PLOS One

Dear Dr. touitou-burckard,

I'm pleased to inform you that your manuscript has been deemed suitable for publication in PLOS One. Congratulations! Your manuscript is now being handed over to our production team.

At this stage, our production department will prepare your paper for publication. This includes ensuring the following:

* All references, tables, and figures are properly cited

* All relevant supporting information is included in the manuscript submission,

* There are no issues that prevent the paper from being properly typeset

You will receive further instructions from the production team, including instructions on how to review your proof when it is ready. Please keep in mind that we are working through a large volume of accepted articles, so please give us a few days to review your paper and let you know the next and final steps.

Lastly, if your institution or institutions have a press office, please let them know about your upcoming paper now to help maximize its impact. If they'll be preparing press materials, please inform our press team within the next 48 hours. Your manuscript will remain under strict press embargo until 2 pm Eastern Time on the date of publication. For more information, please contact onepress@plos.org.

You will receive an invoice from PLOS for your publication fee after your manuscript has reached the completed accept phase. If you receive an email requesting payment before acceptance or for any other service, this may be a phishing scheme. Learn how to identify phishing emails and protect your accounts at https://explore.plos.org/phishing.

If we can help with anything else, please email us at customercare@plos.org.

Thank you for submitting your work to PLOS ONE and supporting open access.

Kind regards,

PLOS ONE Editorial Office Staff

on behalf of

Dr. Eleni Petkari

Academic Editor

PLOS One
